# The General Hospitals of Paris

**Published:** 1867-01

**Authors:** 


*The General Hospitals of Paris.*—These are eight in number, and as a mean, deduced from the experience of eight years (1855–'63), furnished the following statistics: Hôtel-Dieu, 796 beds, with mortality of 10.54 per cent.; Pitié, 594 beds, mortality 11.91; Charité, 480 beds, mortality 9.59; St. Antoine, 330 beds, mortality 10.39; Necker, 346 beds, mortality 11.02; Cochin, 116 beds, mortality 9.85; Beaujon, 330 beds, mortality 11.18; Lariboisière, 617 beds, mortality 11.70.—*New York Med. Jour.*

